# Transfusion-transmitted Syphilis in Teaching Hospital, Ghana

**DOI:** 10.3201/eid1711.110985

**Published:** 2011-11

**Authors:** Alex K. Owusu-Ofori, Christopher M. Parry, Imelda Bates

**Affiliations:** Komfo Anokye Teaching Hospital, Kumasi, Ghana (A.K. Owusu-Ofori); Liverpool School of Tropical Medicine, Liverpool, UK (A.K. Owusu-Ofori, I. Bates); University of Oxford, Oxford, UK (C.M. Parry)

**Keywords:** Treponema pallidum, bacteria, blood transfusion, syphilis, transfusion-transmitted syphilis, serodiagnosis, enzyme immunoassay, screening, prevalence, teaching hospital, sub-Saharan Africa, Ghana

**To the Editor:** Transfusion-transmitted syphilis, which is caused by *Treponema pallidum* subspecies *pallidum*, is one of the oldest recognized infectious risks of blood transfusion (*1*). Routine screening of blood donors and refrigeration of donated blood before its use has resulted in only 3 reported cases of transfusion-transmitted syphilis over the past 4 decades (*2**–**6*).

The World Health Organization recommends screening all donated blood for syphilis (*7*), but doing so is challenging for many developing countries. Many blood banks in low-income countries, including Komfo Anokye Teaching Hospital in Kumasi, Ghana, do not screen donated blood for syphilis.

This study was conducted at Komfo Anokye Teaching Hospital. The purpose of this study was to determine the prevalence of syphilis among blood donors and whether seroconversion occurred in transfusion recipients. The study was approved by the ethics committees in Kumasi, Ghana, and Liverpool, UK.

Pretransfusion plasma samples from 200 conscious transfusion recipients in adult, pediatric, and obstetric inpatient departments and samples of their transfused blood were tested for syphilis. A positive initial result by enzyme immunoassay (EIA) (Bioelisa Syphilis 3.0; Biokit, Barcelona, Spain) was confirmed by using a *T. pallidum* hemagglutination assay (TPHA) (Syphagen; Biokit). A rapid plasma reagin (RPR) assay (RPR Reditest; Biokit) was used to determine whether seropositivity was caused by recent infection. Seronegative recipients who had received seropositive blood were retested 30 days posttransfusion to identify seroconversions. All donors and recipients with recent infections were offered counseling and treatment in accordance with national guidelines.

A total of 145 (73%) blood donors were male, and 109 (57%) units of blood had been stored for <4 days. Sixteen units (8%, 95% confidence interval [CI] 4.3%–11.7%) were seropositive for syphilis by EIA and TPHA. Of these units, 7 (44%) were RPR reactive, which indicated a prevalence of recent infections of 3.5% (95% CI 1.0%–6.0%) (Table). Twenty-six transfusion recipients (13%; 95% CI 8.3%–17.7%) were seropositive by EIA and TPHA. Of these recipients, blood samples from 9 (35%) were RPR reactive, indicating a prevalence of recent infection of 4.5%.

**Table:** Characteristics of 16 recipients of syphilis-positive blood transfusions, Kumasi, Ghana*

Recipient ID	RPR results for transfused blood	Duration of blood storage, d	Blood sample test results							Outcome
			Pretransfusion				Posttransfusion			
			EIA	TPHA	RPR		EIA	TPHA	RPR	
1	R	12	–	ND	ND		NA	NA	NA	Died
2	NR	2	–	ND	ND		NA	NA	NA	Died
3	NR	2	–	ND	ND		NA	NA	NA	Died
4	NR	1	–	ND	ND		NA	NA	NA	Died
5	R	4	–	ND	ND		NA	NA	NA	Lost to follow up
6	NR	1	–	ND	ND		NA	NA	NA	Lost to follow up
7	NR	2	+	+	NR		NA	NA	NA	Not followed up
8	NR	6	+	+	R		NA	NA	NA	Not followed up
9	NR	3	–	ND	ND		–	ND	ND	Well
10	R	1	–	ND	ND		+	+	R	Seroconverted
11	NR	2	–	ND	ND		–	ND	ND	Well
12	R	1	–	ND	ND		–	ND	ND	Well
13	R	3	–	ND	ND		+	–	NR	Well
14	NR	2	–	ND	ND		–	ND	ND	Well
15	R	1	–	ND	ND		–	ND	ND	Well
16	R	4	–	ND	ND		–	ND	ND	Well

*ID, identification; RPR, rapid plasma reagin; EIA, enzyme immunoassay; TPHA, *Treponema pallidum* hemagglutination assay; R, reactive; –, negative; ND, not done; NA, not available; NR, not reactive; +, positive. All results for transfused blood tested by EIA and TPHA were positive.

One recipient, an 8-year-old girl with severe malarial anemia (recipient 10), showed seroconversion after receiving an RPR-reactive unit of blood that had been refrigerated for only 1 day before being issued for use. Posttransfusion fever developed in this recipient, who responded to treatment with cefuroxime and gentamicin, although results of blood culture for bacteremia and peripheral blood film for malaria parasites were negative. She had no relevant sexual history, had been febrile after the transfusion, and showed no evidence of mucocutaneous lesions or lymphadenopathy at her follow-up visit 1 month after the transfusion. She was referred to pediatricians for treatment of syphilis.

This recipient who showed seroconversion most likely had a case of transfusion-transmitted syphilis. Other treponemal infections such as yaws cannot be differentiated serologically from syphilis, and a diagnosis of yaws is based on clinico-epidemiologic features (*8*); however, yaws is not endemic to Kumasi, and because this child had no clinical evidence of yaws, this disease is unlikely to be the cause of the seroconversion.

Refrigeration of units of blood for ≥5 days kills *T*. *pallidum*, but 57% of the donated blood in this study was stored for <4 days before use. This situation prevails across many blood banks in sub-Saharan Africa where, because of inadequate supply and high demand, blood is used as soon as it becomes available. Such short periods of blood storage do not provide an adequate margin of safety against transfusion-transmitted syphilis. Findings from this study have been discussed with the hospital transfusion committee, and new syphilis screening guidelines and testing algorithms are being developed.

The high prevalence of syphilis seropositivity in blood donors and seroconversion of a transfusion recipient shows that in centers where screening is not conducted, recipients of blood transfusions are at risk for contracting transfusion-transmitted syphilis. This finding is likely in blood banks that have a high demand for blood and where blood is stored only for a few days. This study highlights transfusion-transmitted syphilis as a serious public health issue in developing countries and demonstrates that screening of donor blood for syphilis should be conducted.
